# Epidemiological dynamics and early warning of norovirus and rotavirus A in Yantai City in 2023-2024 based on wastewater surveillance

**DOI:** 10.3389/fmicb.2025.1761343

**Published:** 2026-01-16

**Authors:** Shicui Yan, Guofeng Xu, Xuebin Ding, Lili Zhao, Qiao Gao, Cong Li, Hongtao Wang, Zexin Tao, Zhenlu Sun

**Affiliations:** 1 Yantai Center for Disease Control and Prevention, Yantai, Shandong, China; 2 Yantai Zhifu District Center for Disease Control and Prevention, Yantai, Shandong, China; 3 Shandong Center for Disease Control and Prevention, Jinan, Shandong, China

**Keywords:** cross-correlation analysis, Multiplex RT-qPCR, norovirus (NoV), rotavirus A (RVA), wastewater-based epidemiology (WBE)

## Abstract

**Objective:**

The aim is to address the limitations of clinical surveillance—specifically, its high cost and underreporting of asymptomatic infections and untreated individuals—by implementing municipal wastewater surveillance. This study characterizes the epidemiological dynamics of Norovirus (NoV) and Rotavirus A (RVA) in Yantai City and evaluates the effectiveness of Wastewater-Based Epidemiology (WBE) for early outbreak warning.

**Methods:**

From 2023 to 2024, weekly wastewater samples (1–2 samples per site) were collected from 10 municipal wastewater treatment plants (WWTPs) across five urban districts and three counties in Yantai City. Following concentration via polyethylene glycol (PEG) precipitation, viral nucleic acids of NoV GI/GII and RVA were examined using multiplex reverse transcription quantitative PCR (RT-qPCR), with quantification based on standard curves. Cross-correlation analysis was applied to assess time-lag relationships between viral concentrations in wastewater and clinical case peaks, and to evaluate the statistical significance (α = 0.05) of the early warning time window.

**Results:**

Wastewater surveillance (no. samples: 1,391) identified NoV GII as the dominant virus with an overall detection rate of 85.84%. However, from 2023 to 2024, its annual detection rate declined significantly from 92.27% to 73.81% (*P* < 0.001). During the same period, NoV GI also declined annually from 83.55% to 69.07% (*P* < 0.001), whereas RVA detection increased substantially by 145.7% annually, rising from 26.6% to 65.36% (*P* < 0.001). NoV peaked in winter-spring seasons (GI: 76.61% in winter, 89.20% in spring; GII: 87.13% in winter, 91.31% in spring), whereas RVA peaked in spring (42.72%) and summer (55.79%). Seasonal fluctuation intensity followed this order: RVA (χ^2^ = 69.07) > NoV GI (χ^2^ = 49.28) > NoV GII (χ^2^ = 21.44). Cross-correlation analysis indicated that NoV GII concentration in wastewater peaked 1 month ahead of clinical cases, showing significant positive correlations with both reported cases (*r* = 0.60, *P* = 0.002) and clinical positivity rates (*r* = 0.53, *P* = 0.009) at a one-month lag. A one-month lag for NoV GI and a two-month lag for RVA relative to clinical cases were observed but were not statistically significant (*P* > 0.05).

**Conclusion:**

Systematic wastewater surveillance effectively captured population-level epidemiological dynamics of NoV and RVA. Notably, NoV GII provided a significant one-month early warning signal (*P* < 0.01), establishing its value as a leading indicator for diarrheal virus prevention and control in Yantai City.

## 1 Introduction

Norovirus (NoV) and rotavirus (RV) are the primary etiological agents of viral gastroenteritis worldwide, responsible for both sporadic cases and outbreak events (Ahmed et al., 2014; Carlson et al., 2024; Wang et al., 2025; Zhang et al., 2022). NoV imposes the most substantial disease burden, causing approximately 685 million annual cases globally with over 200,000 attributable deaths (including an estimated 70,000 deaths among children under 5 years), and resulting in economic losses estimated at US$60 billion (encompassing healthcare expenditures and productivity losses) (Carlson et al., 2024; Kirk et al., 2015; Lopman et al., 2016). This burden disproportionately affects young children and the elderly. Genetically, NoV is classified into 10 genogroups (GI-GX) and 49 genotypes based on capsid protein sequence variations (Chhabra et al., 2019). Human infections are predominantly caused by GII, followed by GI and GIV. RV poses a significant global health threat. In 2016, RV caused over 258 million infections in children under five worldwide. This pathogen accounted for approximately 500,000 deaths in this age group in 2019, with RV-associated diarrheal mortality remaining substantial at 170,000 deaths globally in 2021 (Wang et al., 2025). Despite the inclusion of RV vaccines in national immunization programs (NIPs) of numerous countries since 2006, vaccine efficacy demonstrates significant geographical heterogeneity. Low-income countries continue to bear a heavy disease burden (Du et al., 2022; Kyu et al., 2025; Stanifer and Boulant, 2020; Vetter et al., 2021). Rotaviruses are classified into seven groups (A-G) based on antigenic differences in the VP6 capsid protein. Among these, rotavirus A (RVA) represents the primary human pathogen and serves as the core target of current vaccination strategies (Sun et al., 2024). Therefore, sustained surveillance of both NoV (particularly GI/GII) and RV (specifically RVA) remains critically important for public health protection.

Current clinical surveillance relies on samples from healthcare institutions, making it difficult to capture mild and asymptomatic infections. Evidence indicates that NoV and RVA cause a high proportion of asymptomatic acute gastroenteritis (AGE) cases in communities (Quee et al., 2020; Teunis et al., 2014). Surveillance in China faces additional challenges: the absence of systematic surveillance with laboratory-confirmed etiology results in most sporadic cases lacking confirmed etiological identification. At the same, ongoing viral evolution necessitates comprehensive tracking of epidemiological trends and emergent strains to support early warning systems. Wastewater, serving as an integrated sample of viral excretion from populations, provides a unique perspective for monitoring the population-level transmission dynamics of diarrheal viruses such as NoV and RVA, thereby serving as a powerful tool for community-wide surveillance. While traditional infectious disease surveillance methods—such as sentinel surveillance, hospital data analysis, and questionnaires—remain effective, they often incur high costs and require specialized personnel. Their efficacy is susceptible to data quality limitations, including misdiagnosis, underreporting, healthcare-seeking behavior, and healthcare accessibility constraints (Mao et al., 2020). As a critical complementary tool for understanding population-level transmission dynamics, wastewater surveillance substantially mitigates these limitations of conventional clinical monitoring through its broad coverage, high cost-effectiveness, and strong objectivity. It rapidly and reliably provides population health data unaffected by healthcare-seeking behavior, while its high sensitivity enables early detection of pathogen transmission to facilitate timely community-based control strategies (Xagoraraki and O’Brien, 2020).

As an ideal tool for monitoring viral prevalence, wastewater surveillance has been successfully applied to detect diverse pathogens including poliovirus, SARS-CoV-2, and influenza viruses (O’Brien and Xagoraraki, 2019). Its core value lies in providing unbiased population-level estimates of disease prevalence. For poliovirus, such surveillance serves as a critical supplementary strategy within the Global Polio Eradication Initiative (GPEI) and has been incorporated into World Health Organization (WHO) guidelines (Dept et al., 2013). Ongoing environmental surveillance for poliovirus and enteroviruses in Shandong Province has provided critical evidence for virus tracing and early warning (Wang et al., 2014). During the COVID-19 pandemic, wastewater surveillance was globally deployed for community-level SARS-CoV-2 screening, demonstrating its capacity not only for early detection of localized outbreaks (Chavarria-Miró et al., 2021; Deng et al., 2022; Medema et al., 2020; Zheng et al., 2022), but also for revealing significant temporal correlations between viral concentrations in sewage and clinically confirmed case numbers (Daughton, 2020; Peccia et al., 2020; Weidhaas et al., 2021; Wurtzer et al., 2020). Studies confirm that both NoV and RV are shed in the feces of symptomatic and asymptomatic individuals. Given that viral diarrhea is primarily transmitted via the fecal-oral route, with both patients and asymptomatic carriers exhibiting persistent viral shedding for weeks, this provides a theoretical foundation for wastewater-based surveillance (WBS) of viral gastroenteritis (Zeng et al., 2019). Furthermore, after entering wastewater through infected feces, host-specific NoV and RV still maintain high concentrations. Since these viruses are incapable of replication outside hosts, the viral concentration in raw sewage theoretically directly reflects the local burden of AGE infections within the population.

To assess the significance of wastewater surveillance, elucidate the distribution patterns and epidemic trends of diarrheal viruses in Yantai, and provide evidence-based support for establishing disease control and early warning systems, this study employed multiplex real-time reverse transcription quantitative PCR (RT-qPCR) to analyze 1,391 samples collected from ten wastewater treatment plants (WWTPs) across five urban districts and three counties of Yantai between 2023 and 2024. The research focused on the overall detection rates and temporal trends (annual, monthly, and quarterly) of NoV GI/GII and RVA. Time-series correlation analysis between diarrheal virus concentrations in wastewater and clinically confirmed cases or patient positivity rates were conducted through cross-correlation analysis to establish early warning time windows. It provides early warning signals and identifies cryptic transmission chains with asymptomatic infections, enabling real-time prediction of epidemic trends and estimation of actual infection magnitude. This facilitates proactive containment of transmission dynamics, significantly reducing the disease burden.

## 2 Materials and methods

### 2.1 Samples and data sources

This study collected wastewater samples from ten representative WWTPs across Yantai City from January 2023 to December 2024. The study was conducted in accordance with the Declaration of Helsinki and approved by the Ethics Committee of Yantai Center for Disease Control and Prevention (No: YYLLS 2023-30). These sites covered five urban districts (Zhifu, Kaifa, Muping, Fushan, and Penglai) and three county-level cities (Zhaoyuan, Longkou, and Haiyang). Clinical case data were obtained from Yantai Center for Disease Control and Prevention (CDC) viral diarrhea etiological surveillance network, including reported NoV and RVA infections from sentinel hospitals, and pathogen-positive cases identified in fecal specimens collected by district CDCs. Positive detection rates were calculated as: number of positive cases/total tested samples × 100%.

### 2.2 Surveillance subjects and sampling frequency

Wastewater samples in this study were collected from January 15, 2023 to December 26, 2024, with the following protocol: During 2023 (January 15–December 28), two discrete samples per week were collected from each WWTP, with sampling conducted every Monday and Thursday. This yielded 906 valid samples annually. During 2024 (January 2–December 26), the sampling frequency was adjusted to one discrete sample per week, collected every Tuesday, resulting in 485 valid annual samples.

### 2.3 Sample collection, preservation, and transportation

Untreated raw wastewater was collected from the influent channel of WWTPs using an automatic sampler. Time-proportional composite sampling was performed: from 10:00 to 10:00 the next day, 100 mL were collected hourly and mixed into a 24-hour composite sample (∼ 2400 mL of total volume). Immediately after collection, samples were transferred to pre-cooled (4°C) sampling bottles sterilized with 75% ethanol, and transported within 2 h to the Yantai Laboratory for Virological Diarrhea Pathogen Detection using dedicated cold-chain containers (0–4°C).

### 2.4 Wastewater sample processing and virus concentration

The wastewater sample underwent a three-step concentration procedure: (1) Pre-centrifugation for Debris Removal: 45 mL of sample was centrifuged at 2,000 × *g* for 2 min at 4°C. The supernatant (40 mL) was collected. (2) PEG-NaCl Precipitation: Polyethylene glycol 8000 (PEG-8000, 4.0 ± 0.1 g) (Shanghai Aladdin Biochemical Technology Co., Ltd., China) and Sodium chloride (NaCl, 0.8 ± 0.01 g) were added to a new centrifuge tube. The 40 mL supernatant was transferred to this tube. The mixture was shaken at 150 × g for 120 min at 4°C, followed by centrifugation at 4,800 × *g* for 45 min at 4°C. The supernatant was discarded. The pellet was thoroughly resuspended in the remaining 1 mL of liquid. (3) Virus Concentration: The resuspended solution was transferred to a 1.5 mL microcentrifuge tube and centrifuged at 20,000 × *g* for 8 min at 4°C. The supernatant was discarded. The pellet was resuspended in the remaining 500 μL of liquid and vortexed thoroughly, generating the concentrated sample.

### 2.5 Nucleic acid extraction and detection

Samples were processed within 24 h of receipt using the following protocol: (1) Nucleic Acid Extraction: Viral nucleic acids were extracted from the wastewater concentrate using the fully automated nucleic acid extraction system and its accompanying magnetic bead-based kit (Jiangsu Bioperfectus Technologies Co., Ltd., China). (2) Multiplex Real-Time RT-qPCR: Amplification was performed using the RVA/Nov GI/Nov GII nucleic acid detection kit (BioGerm Medical Technology Co., Ltd., China). Each 25 μL reaction composition contained: 7.5 μL Reaction Mix, 7.5 μL RVA/NoV GI/NoV GII Primer-Probe Mix, 5 μL Enzyme Mix, and 5 μL template nucleic acid. Amplification Protocol: The thermal cycling conditions were: reverse transcription at 50°C for 10 min → pre-denaturation at 95°C for 5 min → 40 cycles of amplification (denaturation: 95°C for 10 s; annealing/extension: 55°C for 40 s). Result Interpretation Criteria: Positive for NoV GI: FAM channel Ct value ≤38; Positive for NoV GII: VIC channel Ct value ≤38; Positive for RVA: CY5 channel Ct value ≤38. Samples with a Ct value >38 but ≤40 for any target were retested. Samples were considered positive upon retest if the Ct value remained ≤40 and exhibited a characteristic amplification curve. Samples with Ct values >40 were considered negative. (3) Quality Control: Appropriate positive and negative controls were included in each assay.

### 2.6 Construction of standard curves for viral quantification

Standard curves were constructed using certified nucleic acid standards for NoV GI/GII and RVA (Engineering Research Center of Industrial Microorganisms, Hebei Province, China). Serial 10-fold dilutions of the standards (ranging from 10^4^ to 10^7^ copies/mL) were prepared. For validation, the standard curves were required to meet both of the following criteria: Amplification eff.iciency (E) ideally between 90% and 110%, calculated as: E = [10^(–1/*slope*)^–1] × 100%; Linear correlation coefficient (R^2^) ≥ 0.99.

### 2.7 Calculation of viral nucleic acid concentration

The concentrations of NoV GI/GII and RVA nucleic acids in wastewater were calculated using the following formula: (E × D × B)/(A × C). Where the parameters are defined as: A: Volume of raw wastewater sample processed prior to concentration (mL); B: Final volume of the virus concentrate after reconstitution (mL); C: Volume of virus concentrate used for nucleic acid extraction (μL); D: Elution volume of extracted nucleic acids (μL); E: Viral genome concentration in the nucleic acid extract determined by the standard curve (copies/mL).

### 2.8 Statistical analysis

Data were processed and summarized using Microsoft Excel 2010. Statistical analyses were performed with SPSS 30.0. Chi-square (χ^2^) tests were applied to compare proportions; Fisher’s exact tests were substituted when expected frequencies were <5. Statistical significance was defined as *P* < 0.05. Cross-correlation analysis was conducted in R 4.4.2 using the ggplot2 (v3.4.4) and ggpubr (v0.6.0) packages to examine time-lagged relationships between viral concentrations in wastewater (copies/mL) and clinically confirmed cases or positivity rates with the following specifications: (1) Analysis period: January 2023–December 2024 (*n* = 24 monthly data points); (2) Lag range: k ∈ [-2, 2] months, where: k > 0: Wastewater data leading clinical data; (3) Correlation method: Pearson’s *r* coefficients and corresponding *P*-values were computed at each lag interval. 4) Significance criteria: Moderate correlation: 0.4 ≤ | *r*| < 0.6 with *P* < 0.05; Strong correlation: 0.6 ≤ | *r*| < 0.8 with *P* < 0.05; 5) Visualization: Cross-correlation functions were examined through lag plots and bivariate time-series plots.

## 3 Results

### 3.1 Overall detection characteristics of NoV and RVA in wastewater

From January 2023 to December 2024, a total of 1,391 wastewater samples were collected from Yantai City. Multiplex RT-qPCR analysis revealed NoV was predominantly detected with the highest prevalence for GII (85.84%, 1194/1391), followed by GI (78.50%, 1092/1391). The co-detection rate for both GI and GII reached 76.06% (1058/1391). In contrast, the overall detection rate of RVA was relatively low at 40.12% (558/1391). Concurrently, the co-detection rate for multiple viruses (RVA and NoV GI/GII) was 35.37% (492/1391). Detailed distributions are presented in Table 1.

**TABLE 1:** Overall detection profile of RVA and NoV GI/GII in wastewater of Yantai City, 2023–2024.

Year	Sample size	RVA positive		NoV GI positive		NoV GII positive		NoV GI/GII positive		RVA and NoV positive	
		No.	%	No.	%	No.	%	No.	%	No.	%
2023	906	241	26.60	757	83.55	836	92.27	734	81.02	227	25.06
2024	485	317	65.36	335	69.07	358	73.81	324	66.80	265	54.64
Total	1391	558	40.12	1092	78.50	1194	85.84	1058	76.06	492	35.37

### 3.2 Annual and seasonal distribution characteristics of viral detection rates in wastewater

#### 3.2.1 Annual variations

Wastewater surveillance in Yantai City during 2023–2024 demonstrated that NoV GII remained the predominant pathogen. However, its detection rate significantly decreased from 92.27% to 73.81% (χ^2^ = 88.54, *P* < 0.001), while NoV GI declined from 83.55% to 69.07% (χ^2^ = 39.26, *P* < 0.001). In contrast, RVA detection rates substantially increased from 26.60% to 65.36% (χ^2^ = 197.56, *P* < 0.001). χ^2^ tests confirmed statistically significant differences in detection rates across years for all three viruses, with RVA exhibiting the most pronounced magnitude of change—a 145.7% increase. Detailed results are presented in Table 2.

**TABLE 2:** Annual Detection Variations of RVA and NoV GI/GII in wastewater of Yantai City, 2023–2024.

Year vs detection profile	2023	2024	Magnitude of change	Rate of increase/decrease	χ^2^ value	*P-*value
Total samples	906	485	\	\	\	\
RVA positive samples (detection rate)	241 (26.60%)	317 (65.36%)	+38.76%	+145.7%	197.56	<0.001
NoV GI positive samples (detection rate)	757 (83.55%)	335 (69.07%)	−14.48%	−17.33%	39.26	<0.001
NoV GII positive samples (detection rate)	836 (92.27%)	358 (73.81%)	−18.46%	−20.00%	88.54	<0.001

Magnitude of Change = (detection rate in 2024 - detection rate in 2023); Rate of change (%) = (Magnitude of Change/detection rate in 2023) × 100%; Rate of change covers both rate of increase and rate of decrease.

#### 3.2.2 Monthly and seasonal dynamics

**FIGURE 1:**
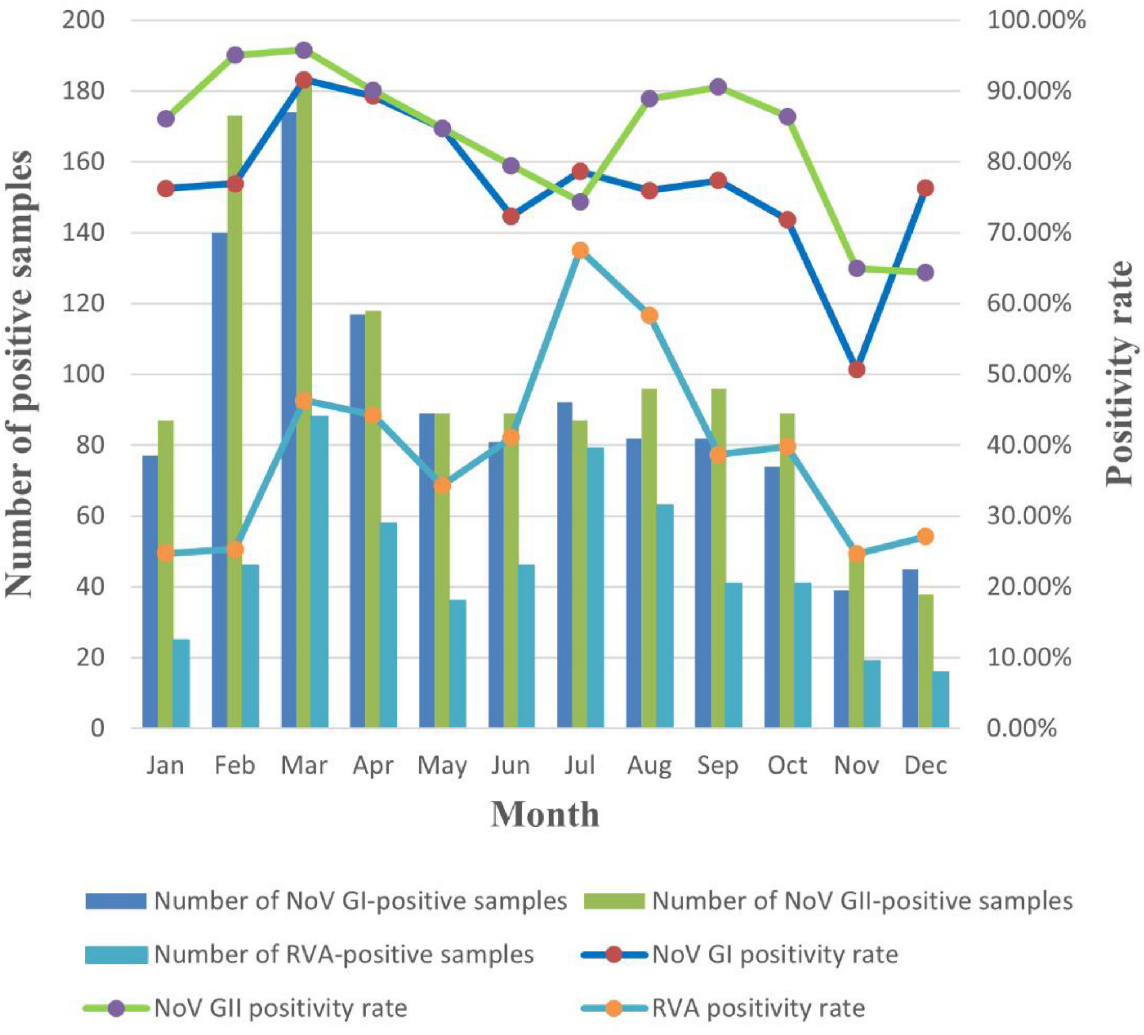
Monthly distribution of RVA and NoV GI/GII in wastewater of Yantai City, 2023–2024.

Viral detection in this study exhibited persistent monthly distribution patterns and significant seasonal fluctuations. Both RVA and NoV GI/GII were detected year-round, with NoV GI/GII peaking in March (GI: 91.58%; GII: 95.79%) and reaching nadirs in November (50.65%) and December (64.41%), respectively. RVA also showed substantial monthly variations, peaking in July (67.52%) and declining to its lowest level in November (24.68%) (Figure 1). Seasonal analysis revealed statistically significant variations for all three viruses (χ^2^ tests, all *P* < 0.001). NoV GI/GII demonstrated winter-spring predominance (GI: 76.61% in winter, 89.20% in spring; GII: 87.13% in winter, 91.31% in spring), whereas RVA peaked during spring-summer (42.72% in spring, 55.79% in summer). Comparison of seasonal variation magnitudes based on χ^2^ values ranked as follows: RVA > NoV GI > NoV GII, with RVA exhibiting the most pronounced seasonal variation (χ^2^ = 69.07). Detailed results are presented in Table 3.

**TABLE 3:** Seasonal detection variations of RVA and NoV GI/GII in wastewater of Yantai City, 2023–2024.

Season vs detection rate	NoV GI detection rate (positive/total quarterly samples)	NoV GII detection rate (positive/total quarterly samples)	RVA detection rate (positive/total quarterly samples)
Spring (Mar, Apr, May)	89.2% (380/426)	91.31% (389/426)	42.72% (182/426)
Summer (Jun, Jul, Aug)	75.67% (255/337)	80.71% (272/337)	55.79% (188/337)
Autumn (Sep, Oct, Nov)	68.18% (195/286)	82.17% (235/286)	35.31% (101/286)
Winter (Dec, Jan, Feb)	76.61% (262/342)	87.13% (298/342)	25.44% (87/342)
χ^2^ value	49.28	21.44	69.07
*P-*value	<0.001	<0.001	<0.001

### 3.3 Temporal association analysis between diarrheal virus concentrations in wastewater and clinical case detection

#### 3.3.1 NoV GI/GII

**FIGURE 2:**
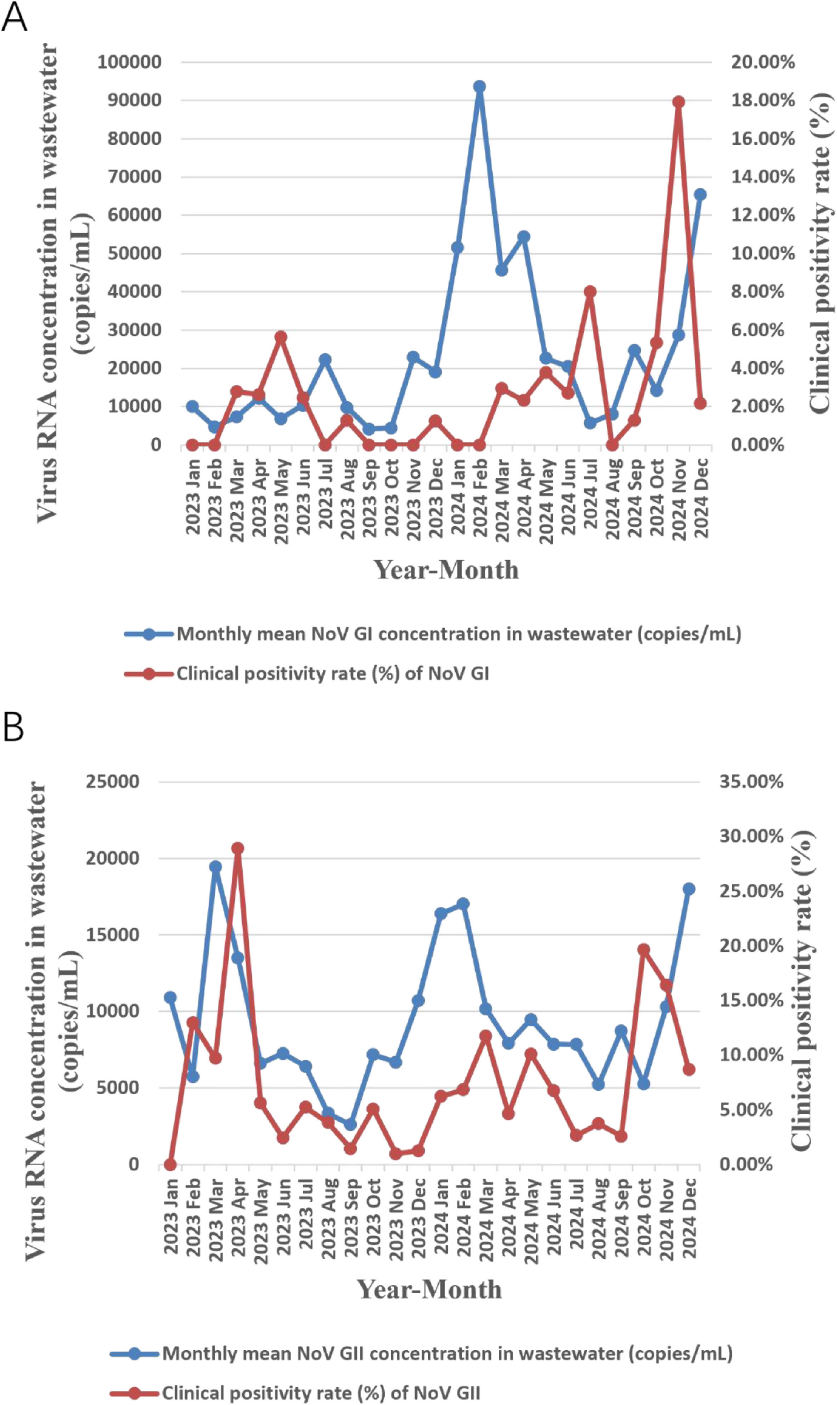
Temporal association analysis between monthly mean nucleic acid concentration of NoV GI **(A)** and GII **(B)** in wastewater and the clinical case positivity rate.

Surveillance data from January 2023 to December 2024 revealed periodic fluctuations in nucleic acid concentrations of both NoV GI and GII in wastewater. Peak concentrations of NoV GI occurred in April, July, and November 2023, as well as February, April, and June 2024; NoV GII peaks were observed in January, March, and June 2023, as well as February, July, and September 2024. Correspondingly, clinical surveillance revealed that the monthly positive detection rates for NoV GI and GII exhibited temporal change patterns significantly similar to those of viral concentrations in wastewater. Critically, clinical case peaks systematically lagged behind virus concentration peaks in wastewater by approximately 1 month: NoV GI clinical peaks occurred in May, August and December 2023, as well as March, May, and July 2024; NoV GII clinical peaks were observed in February, April and July 2023, as well as March, August, October 2024. These findings indicated that monitoring NoV GI/GII nucleic acid concentrations in wastewater provides a one-month early warning indicator for population infection dynamics. Detailed results are shown in Figure 2.

#### 3.3.2 RVA

**FIGURE 3:**
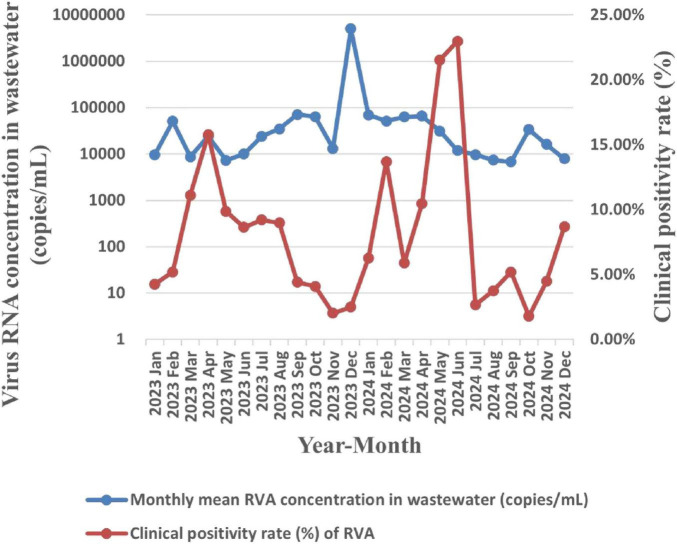
Temporal association analysis between monthly mean nucleic acid concentration of RVA in wastewater and the clinical case positivity rate.

Synchronous wastewater surveillance data revealed periodic fluctuations in RVA nucleic acid concentrations, with peak levels occurring in February and December 2023, as well as April and October 2024. In clinical case surveillance, the monthly positivity rate of RVA demonstrated a parallel trend with viral concentrations detected in wastewater. However, a longer lag time of approximately 2 months was observed for the peak of RVA clinical cases compared to NoV, manifesting specifically in April 2023, as well as February, June and December 2024. These findings indicated that wastewater surveillance holds predictive value for community RVA transmission trends, albeit with an early-warning window of 2 months. Detailed results are shown in Figure 3.

#### 3.3.3 Cross-correlation analysis between viral concentrations in wastewater and clinical cases

**FIGURE 4:**
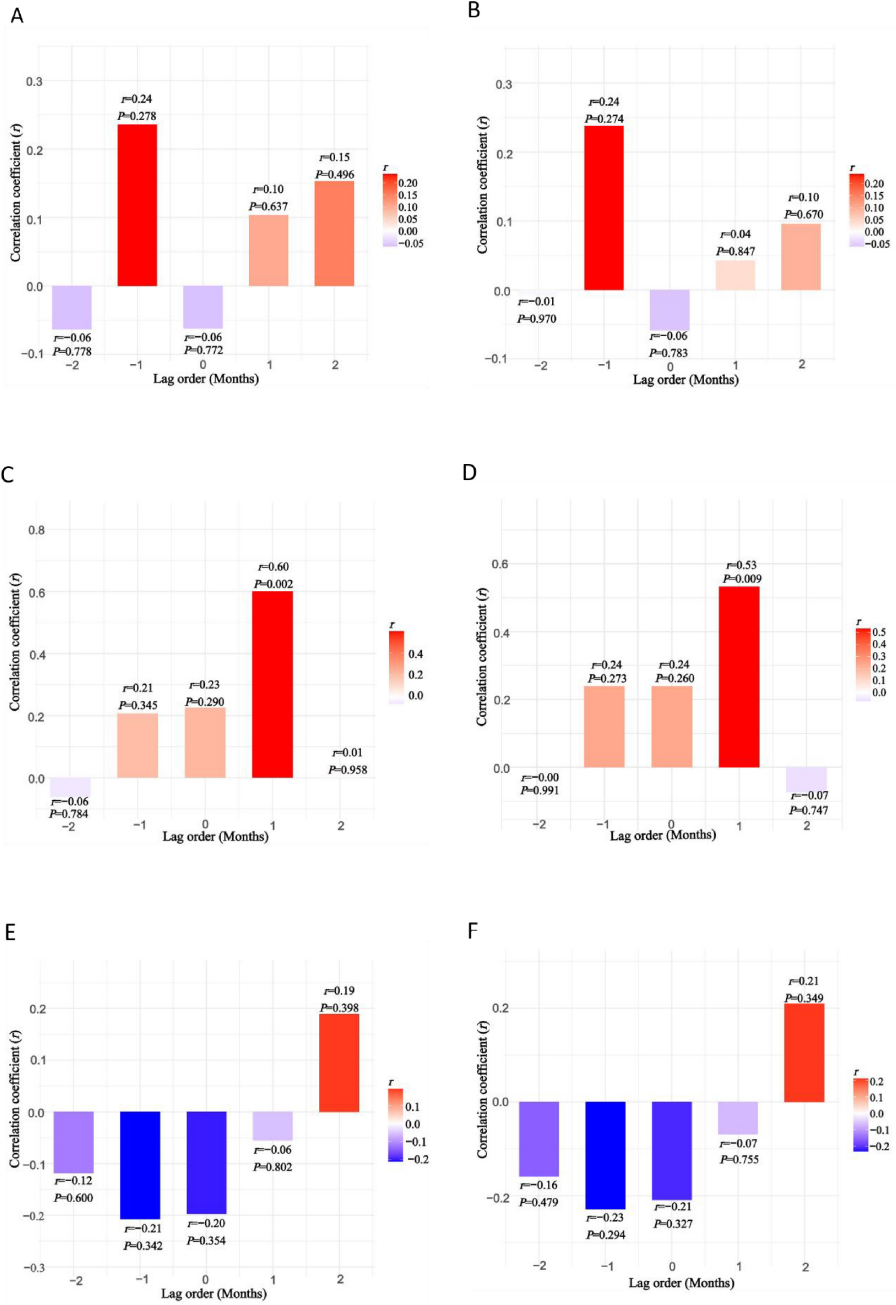
**(A,B)** Cross-correlation analysis between monthly average concentrations of NoV GI in wastewater and clinically confirmed cases or positive detection rates; **(C,D)** cross-correlation analysis between monthly average concentrations of NoV GII in wastewater and clinically confirmed cases or positive detection rates; **(E,F)** cross-correlation analysis between monthly average concentrations of RVA in wastewater and clinically confirmed cases or positive detection rates.

To quantify the lead-lag relationships and assess statistical significance between viral concentrations in wastewater and clinical cases, cross-correlation analysis was performed (Figure 4). The results revealed significant positive correlations between NoV GII concentrations in wastewater and monthly confirmed clinical cases lagged by 1 month (*r* = 0.60, *P* = 0.002) or clinical positivity rates lagged by 1 month (*r* = 0.53, *P* = 0.009). These statistical findings confirmed that NoV GII signals in wastewater stably led clinical case peaks by approximately 1 month, suggesting that wastewater surveillance could provide early warnings for community NoV GII outbreaks 1 month in advance. In contrast, no statistically significant correlations were observed at any tested lag intervals (including one and 2 months) between concentrations of either NoV GI or RVA in wastewater and monthly confirmed clinical cases or clinical positivity rates (all *P* > 0.05).

## 4 Discussion

Wastewater-based surveillance (WBS) serves as a valuable routine passive screening tool at the community level, demonstrating significant utility in revealing pre-symptomatic and asymptomatic states of infectious diseases. By analyzing the dynamic distribution of NoV GI/GII and RVA in Yantai’s municipal wastewater during 2023–2024, this study provides the first systematic environmental characterization of local diarrheal virus epidemiology. Key findings include: (1) The persistently high detection rates of NoV (particularly GII) and RVA indicated substantial unreported cases within the community, quantifying the hidden infection burden; (2) While NoV GI/GII detection rates exhibited an annual decline, RVA showed a significant upward trend—signaling a restructuring of local viral dominance; (3) Distinct seasonal divergence emerged: RVA peaked in spring-summer (peaking in July) versus NoV in winter-spring (peaking in March), offering new targets for precision-based interventions; (4) Elevated wastewater viral concentrations, particularly for NoV GII, served as a leading indicator, preceding clinical outbreaks by approximately 1 month.

The wastewater surveillance results indicated a persistently high detection rate of NoV GI/GII and RVA during 2023–2024. This suggests a high prevalence of infections within the population, aligning closely with Gao et al. (2023), who reported that RV and NoV collectively accounted for over 60% of pathogens identified in clinical infectious diarrhea cases in Yantai. This pattern is also highly consistent with epidemiological characteristics observed in other regions across China (Zhang and Zhang, 2017). Notably, NoV GII exhibited a clear dominance in wastewater (85.84%), exceeding both NoV GI (78.50%) and RVA (40.12%). This aligns with global wastewater surveillance studies (Huang et al., 2022) and reinforces the significant disease burden associated with NoV worldwide. However, this contrasts with previous reports (Sun et al., 2024; Zhang and Zhang, 2017) suggesting RVA was the predominant pathogen followed by NoV. This discrepancy may arise from two factors: firstly, NoV demonstrates significantly higher environmental stability and subclinical infection rate in adults, contributing to its elevated detection in wastewater; secondly, the widespread RVA vaccination may be shifting the epidemiological landscape, with NoV potentially emerging as the dominant pathogen. Previous research (Grassly et al., 2025) has demonstrated that wastewater surveillance can concurrently track multiple pathogens. Its capacity for multi-virus co-detection in a single assay enables the identification of potential mixed infection patterns even before clinical symptoms manifest. Compared to clinical surveillance, an integrated multi-pathogen testing approach offers significant cost advantages. In this study, the co-detection rate of NoV GI/GII and RVA reached 35.37%, reflecting the widespread exposure to multiple pathogens within the population. This concurrent circulation of NoV GI/GII and RVA, and likely frequent co-infections with multiple viruses in individuals increases the risk of severe illness, particularly among the elderly, young children, and immunocompromised individuals with chronic conditions. Wastewater surveillance can provide early warnings regarding the diversity of infections within communities, enabling more effective prevention of severe outcomes.

Notably, against the backdrop of the severe global spread of NoV, this study demonstrated a significant annual decline in NoV GI and GII detection rates (*P* < 0.05), potentially reflecting natural cyclic fluctuations or strain variation and replacement. Confirmation of whether this represents a potential “new normal” requires ongoing surveillance for ≥5 years. Concurrently, in contrast to previous research findings of reduced RVA burden following vaccination (Du et al., 2022), this study revealed a significant annual upward trend in the RVA detection rate within wastewater (2023: 26.60% → 2024: 65.36%), representing a growth rate as high as 145.7%. This finding suggests that the local diarrheal pathogen spectrum may be undergoing structural reorganization, and the transmission intensity of RVA within the Yantai community could potentially continue to increase. This phenomenon necessitates monitoring for two potential mechanisms: a decline in childhood RV vaccination coverage leading to a weakened herd immunity barrier, or the emergence of newly circulating strains capable of immune escape.

Previous studies (Ahmed et al., 2013; Tian et al., 2024) have indicated significant seasonal fluctuations in the peak activity of NoV and RVA, characterized by higher prevalence in winter-spring and the lowest levels in summer. Our findings confirmed that NoV peaked during the winter-spring season, reaching its highest level in March, consistent with the traditional epidemic pattern. However, RVA exhibited a spring-summer peak, culminating in July. This pattern differs from the typical winter peak in temperate zones (Omatola and Olaniran, 2022), but aligns with the recent report describing atypical spring-summer epidemic peaks following the COVID-19 pandemic (Li et al., 2025). This phenomenon may be related to the accumulation of susceptible populations after the relaxation of COVID-19 controls, or potentially linked to autoimmune sequelae and complex immune dysregulation (e.g., long COVID) associated with SARS-CoV-2 infection (Guo et al., 2024). Although multiple studies (Sun et al., 2024; Zhang and Zhang, 2017) have shown that RVA clinical cases in temperate regions typically rise in autumn, our wastewater surveillance clearly captured a significant increase in RVA genomic load as early as summer, validating viral dynamics in wastewater as a leading indicator for predicting the onset of autumn clinical epidemics. Previous researches (Martinez et al., 2016; Oh et al., 2021) have demonstrated that while RVA lacks an environmental reservoir, its transmission exhibits significant climatic sensitivity influenced by population density, behavioral patterns, and socioeconomic factors. This often results in rainy season or summer peaks in tropical regions. Entering the late phase of the COVID-19 pandemic in 2023, Yantai City, as a coastal tourist destination, experienced a sharp increase in population density during the summer tourism peak. Frequent group activities (e.g., swimming, communal dining) significantly elevated fecal-oral transmission risks. Concurrently, extreme summer rainstorms potentially caused sewage backup, further augmenting pathogen exposure. Furthermore, existing studies (Amin et al., 2024; Lestari, 2021) indicate that under vaccine-driven selection pressure, RVA has undergone key amino acid substitutions in antigenic sites and genotypic evolution. We speculate that emerging strains may have consequently gained a replication or transmission advantage under high-temperature conditions, a hypothesis warranting detailed genetic sequencing.

Globally, WBS has been implemented in multiple countries (Grassly et al., 2025; Huang et al., 2023) to monitor the concentration and genotype information of diverse enteric viruses, demonstrating robust early-warning capabilities and providing critical insights for predicting epidemic trends in populations. Kazama et al. (2016) observed that NoV GII genomic concentration fluctuated similarly to local AGE case numbers, suggesting an epidemiological association. Wang et al. (2020) further validated, using cross-correlation analysis, that NoV GII peaks in wastewater preceded AGE cases by 2 weeks, confirming its distinct early-warning timeliness. Huang et al. (2022) demonstrated that compared to clinical reporting systems, WBS significantly enhanced the detection sensitivity for prevalent strains. Based on the dynamic changes in NoV GII genomic concentration, effective warnings could be issued 7–28 days prior to the rise in AGE cases. These conclusions were supported by the studies of Mao et al. (2020) and Chacón et al. (2021), both of which confirmed that WBS enabled early warning of AGE epidemics through enteric virus monitoring. Consistent with the above researches, our study visually demonstrated through time-series plots that NoV GI/GII and RVA concentrations changes in wastewater could provide a 1–2 month early warning for clinical epidemics. Further validation via cross-correlation analysis revealed a significant positive correlation between NoV GII concentration changes in wastewater and the number of laboratory-confirmed positive cases (*r* = 0.60, *P* = 0.002) lagged by 1 month or the positivity rate (*r* = 0.53, *P* = 0.009) lagged by 1 month, which quantitatively supported this temporal association. Compared to previously reported warning periods (Huang et al., 2022), this result provided a longer warning window (1 month) for local NoV GII outbreaks in Yantai, offering significant localized early-warning value. For NoV GI and RVA, under the current analytical framework, cross-correlation analysis could not detect a statistically stable lead-lag relationship between viral concentration in wastewater and clinical cases. This did not negate the observed temporal phenomenon in the time-series plots where viral concentration peaks in wastewater preceded clinical case peaks (NoV GI by 1 month, RVA by 2 months). The lack of statistical significance could stem from sample size limitations, strong data noise, non-stationarity, dynamic lag period variation across epidemic seasons, unlike the relatively stable one-month lag observed for NoV GII. Consequently, the temporal association between these viruses (NoV GI, RVA) in wastewater and clinical cases requires further validation through expanded sample sizes and optimized time-series modeling.

WBS’s non-invasive nature circumvents individual privacy issues and lowers ethical risks. Tracking environmental viruses enables rapid assessment of diarrheal pathogens transmission dynamics within communities, providing crucial evidence for outbreak source tracing, risk assessment, and targeted interventions. Two NoV-related gastroenteritis outbreaks in schools in Shandong and Liaoning were traced to contaminated water sources—specifically, a direct drinking water system (Zhou et al., 2015) and a self-supplied well reservoir, respectively. Both epidemics were rapidly contained following source decontamination, highlighting the critical role of water quality monitoring in preventing and controlling NoV outbreaks. This study, based on wastewater surveillance technology, provided a comprehensive analysis of the epidemiological dynamics of NoV and RVA in Yantai City and analyzed the warning value. This approach facilitates real-time tracking of transmission trends for these pathogens across large populations, offering effective early warning capabilities for both endemic spread and outbreaks. Furthermore, this technology can be extended to monitor and track other pathogens and biomarkers, thereby serving as a vital tool for assessing the public health status of Yantai City. As wastewater surveillance technology continues to evolve, it is poised to become a key component of smart city public health systems in the future.

As the pioneering study on diarrheal pathogen dynamics in Yantai City utilizing WBS, these findings provided an important reference for establishing a localized monitoring framework. However, the study has several limitations: First, lack of molecular epidemiological analysis: Only the proportional composition and temporal distribution of viruses in wastewater were described; no viral sequences were obtained, precluding analysis of viral evolutionary characteristics within the covered area. Second, potential overestimation of infection levels: The environmental persistence of non-enveloped viruses, particularly NoV, may lead to periodically elevated concentrations in wastewater monitoring. Third, absence of spatial correlation validation: The analysis focused solely on the temporal association between viral load in wastewater and clinical case numbers; spatial distribution patterns were not assessed. Fourth, short-term data limits trend inference: Data from 2023 to 2024 are insufficient to represent long-term trends; an extended monitoring period is required.

## 5 Conclusion

In conclusion, longitudinal WBS across 10 WWTPs in Yantai City of 2023–2024 analyzed 1,391 samples, elucidating NoV and RVA distinct epidemiological dynamics. The study established WBS as an effective early warning tool, with rising NoV GII concentrations in wastewater preceding clinical case reports by 1 month. Implementing WBS as a complementary surveillance strategy is recommended to enhance early response capacity for enteric pathogen outbreaks in this region.

## Data Availability

The original contributions presented in this study are included in this article/supplementary material, further inquiries can be directed to the corresponding authors.
